# NF2 deficiency accelerates neointima hyperplasia following vascular injury via promoting YAP-TEAD1 interaction in vascular smooth muscle cells

**DOI:** 10.18632/aging.103240

**Published:** 2020-05-18

**Authors:** Xiongshan Sun, Shuang Li, Xueqing Gan, Ken Chen, Dachun Yang, Yongjian Yang

**Affiliations:** 1 Department of Cardiology, The General Hospital of Western Theater Command, Chengdu 610083, China

**Keywords:** NF2, YAP, TEAD1, neointima hyperplasia, vascular restenosis

## Abstract

Neurofibromin 2 (NF2), a potent tumor suppressor, is reported to inhibit proliferation in several cell types. The role of NF2 in neointima hyperplasia after vascular injury is unknown. We explored the role of NF2 in proliferation, migration of vascular smooth muscle cell (VSMC) and neointima hyperplasia after vascular injury. NF2 phosphorylation was elevated in VSMC subjected to platelet-derived growth factor (PDGF)-BB and in artery subjected to vascular injury. Mice deficient for *Nf2* in VSMC showed enhanced neointima hyperplasia after injury, increased proliferation and migration of VSMC after PDGF-BB treatment. Mechanistically, we observed increased nuclear p-NF2, declined p-Yes-Associated Protein (YAP), nuclear translocation of YAP after PDGF-BB treatment or injury. NF2 knockdown or YAP overexpression showed similar phenotype in VSMC proliferation, migration and neointima hyperplasia. YAP inhibition abolished the above effects mediated by NF2 knockdown. Finally, NF2 knockdown further promoted YAP-TEA Domain Transcription Factor 1 (TEAD1) interaction after PDGF-BB treatment. Inhibition of TEAD1 blocked PDGF-BB-induced VSMC proliferation and migration, which were not reversed by either NF2 knockdown or YAP overexpression. In conclusion, NF2 knockdown promotes VSMC proliferation, migration and neointima hyperplasia after vascular injury via inducing YAP-TEAD1 interaction.

## INTRODUCTION

Vascular remodeling is a major cause of various vascular disorders such as in-stent restenosis, atherosclerosis, vein bypass graft failure and transplant vasculopathy [1, 2]. As a process involving a series of pathological changes such as proliferation of vascular smooth muscle cell (VSMC), neointima hyperplasia (or neointima formation) is a major component of remodeling [3, 4]. When suffering from mechanical injury caused by angioplasty or stent deployment, VSMC migrates into the intima and undergoes a transition from contractile to proliferative phenotype [3]. This process leads to vascular restenosis. Underlying mechanisms of neointima hyperplasia are poorly understood. Therefore, elucidating novel etiology of above-mentioned vascular pathological changes is critical for developing effective drugs to prevent against restenosis.

Neurofibromin 2 (*Nf2*, also called merlin) is originally identified as a tumor suppressor factor and involved in various cancers, including schwannoma, meningioma, and malignant mesothelioma [5]. As a widely expressed scaffold-like protein, NF2 is implicated in various cellular processes including proliferation, migration, adhesion and cell-cell contact through intra- and extracellular signal transduction [6]. NF2 exerts its biological roles via interacting with other proteins and initiates subsequent signaling pathways. These pathways include receptor tyrosine kinase, small GTPases, PI3K/Akt, mammalian target of rapamycin and hippo pathways [7]. When phosphorylated on Ser518 in the C-terminal, NF2 would adopt an open, active conformation at the cell membrane and lose its ability to interact with binding partners [8]. NF2 is recently involved in cardiac ischemic/reperfusion injury [9]. However, its functions in vascular diseases such as restenosis are largely unclear.

As a crucial signaling cascade for cell survival and proliferation, hippo consists of several components including Mammalian STE20-like Protein Kinase1/2, Large Tumor Suppressor Homologue1/2 and Yes-Associated Protein (YAP) [10]. YAP is a major effector of hippo pathway and transcriptionally modulates the expression of genes controlling cellular growth [10]. Phosphorylation of YAP creates a binding site for 14-3-3 proteins, which then bind to YAP and prevent nuclear translocation of YAP. Dephosphorylated form of YAP is shutted within nucleus [11]. A recent study also showed that dephosphorylation of NF2 by oxidative stress would induce YAP phosphorylation and exacerbate cardiac ischemic/reperfusion damage [9]. As a transcriptional coactivator, YAP is confirmed to regulate VSMC proliferation, differentiation and migration [12, 13]. Though YAP is also involved in several vascular diseases such as atherosclerosis [14] and pulmonary hypertension [15], little is known about the role of NF2/YAP in vascular restenosis.

In the current study, we demonstrated that NF2 was phosphorylated by injury in vivo and platelet-derived growth factor (PDGF)-BB in vitro. Phosphorylated NF2 was inactive and caused increased proliferation and migration of VSMC via dephosphorylation of YAP. Mice deficient for *Nf2* in VSMC (*Nf2*^flox/flox^sm22cre^+/-^; *Nf2*^-/-^) showed declined YAP phosphorylation, enhanced YAP-TEA Domain Transcription Factor 1 (TEAD1) interaction and aggravated neointima hyperplasia in response to vascular injury. Inhibition of YAP or TEAD1 both abolished the adverse effects of NF2 knockdown. These results provide evidence that phosphorylated NF2 dephosphorylates YAP and induces YAP-TEAD1 interaction, resulting in increased proliferation, migration of VSMC and vascular restenosis.

## RESULTS

### NF2 knockdown aggravates mechanical injury-induced neointima hyperplasia

**Figure 1:**
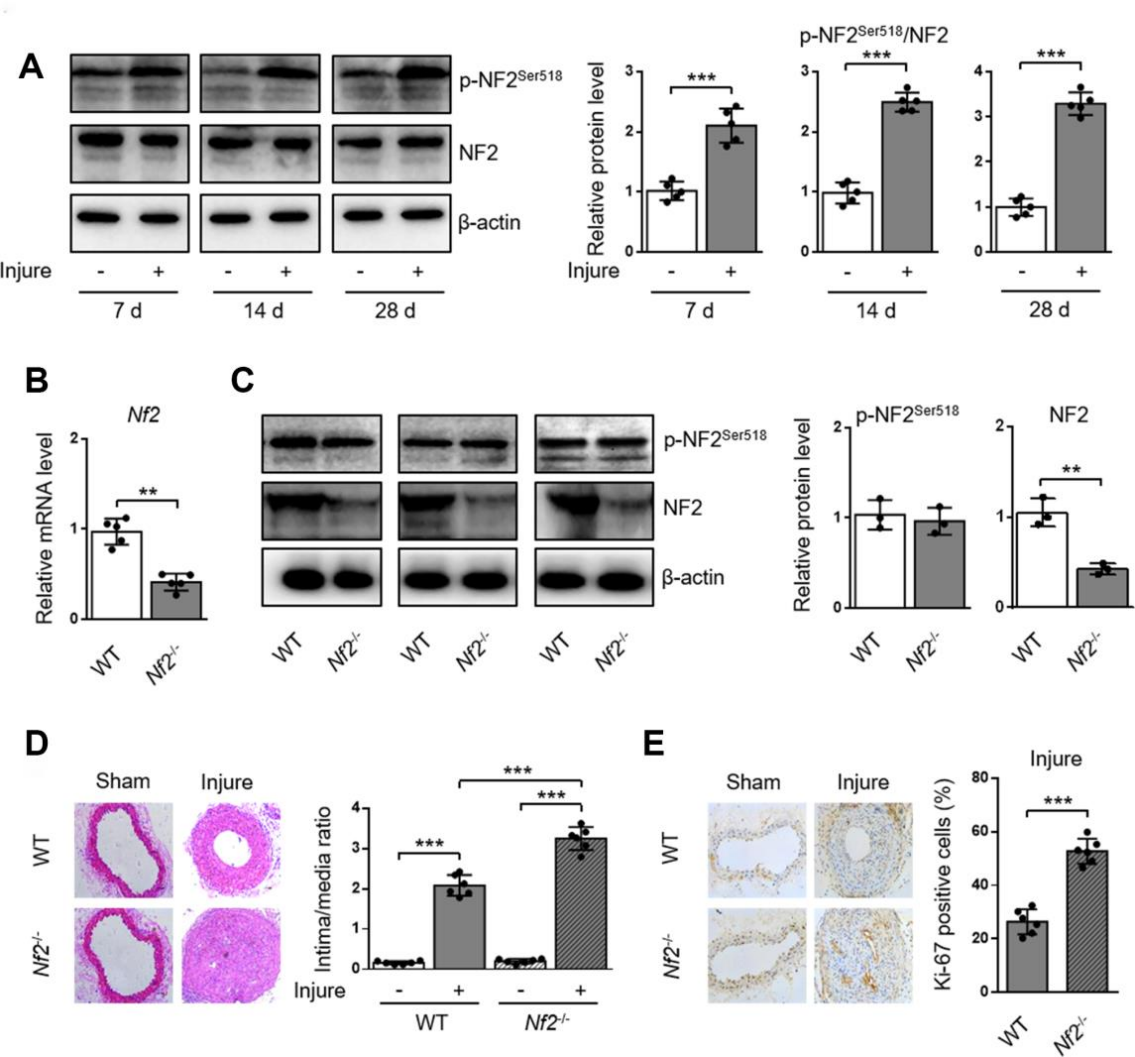
**NF2 knockdown enhances neointima hyperplasia after vascular injury.** WT or *Nf2*^-/-^ mice received sham operation or wire injury in common carotid artery. (**A**) The relative protein levels of p-NF2^Ser518^ and NF2 in common carotid arteries from C57BL/6J mice at day 7, 14 and 28 after injury were analyzed by immunoblotting (n=5). (**B**) The relative mRNA (n=5) level of *Nf2* in common carotid arteries from WT or *Nf2*^-/-^ mice. (**C**) The relative protein (n=3) levels of p-NF2^Ser518^ and NF2 in common carotid arteries from WT or *Nf2*^-/-^ mice. (**D**) Representative H&E staining of carotid arteries from WT or *Nf2*^-/-^ mice at day 28 after sham operation or wire injury (left) and corresponding quantification for ratio of intima/media (right) were shown (n=6). Magnification 200×. (**E**) Immunohistochemistry staining of Ki-67 (brown) in sections of carotid arteries from WT or *Nf2*^-/-^ mice at day 28 after sham operation or wire injury (left) and corresponding quantification for Ki-67 positive cells within neointima (right) were shown (n=6). Magnification 200×. Data are shown as mean ± S.D. *P<0.05, **P<0.01 and ***P<0.001 denote statistical comparison between the two marked groups, respectively.

To investigate whether NF2 is involved in the development of arterial neointima hyperplasia, we first examined the phosphorylation of NF2 in carotid arteries at different time points after injury. Compared with uninjured arteries, protein levels of p-NF2^Ser518^ were more abundant in injured arteries at 7, 14 and 28 days after injury (Figure 1A and Supplementary Figure 1). Given that phosphorylation at Ser518 led to NF2 inactivation [8], we hypothesized that enhanced NF2 phosphorylation (or declined active NF2) might contribute to neointima hyperplasia in response to vascular injury. To determine whether NF2 in VSMC is involved in the development of neointima hyperplasia, we established an animal model in which *Nf2* was knocked down in VSMC (*Nf2*^-/-^). Compared with WT mice, both vascular mRNA (Figure 1B) and protein (Figure 1C, Supplementary Figure 2A and 2B) levels of NF2 significantly declined in *Nf2*^-/-^ mice. Interestingly, NF2 knockdown had little effect on protein expression level of p-NF2^Ser518^ (Figure 1C, Supplementary Figure 2A and 2B). This phenomenon indicates phosphorylation of NF2 may be compensated by certain unknown mechanism after NF2 knockdown. Mechanical injury led to obviously accelerated vascular restenosis, which was reflected by increased intima/media ratio, enlarged Ki-67-positive area and declined mRNA levels of differentiation marker genes (*α-SMA*, *Calponin* and *SM-MHC*) (Supplementary Figure 3) [16] in injured arteries of both WT and *Nf2*^-/-^ mice. Additionally, the degree of neointima hyperplasia was more severe after NF2 knockdown at day 7, 14 and 28 (Figure 1D and 1E; Supplementary Figure 4). These data further suggest a crucial role of NF2 in injury-induced vascular neointima hyperplasia.

### NF2 knockdown enhances proliferation and migration of VSMC induced by PDGF-BB

**Figure 2:**
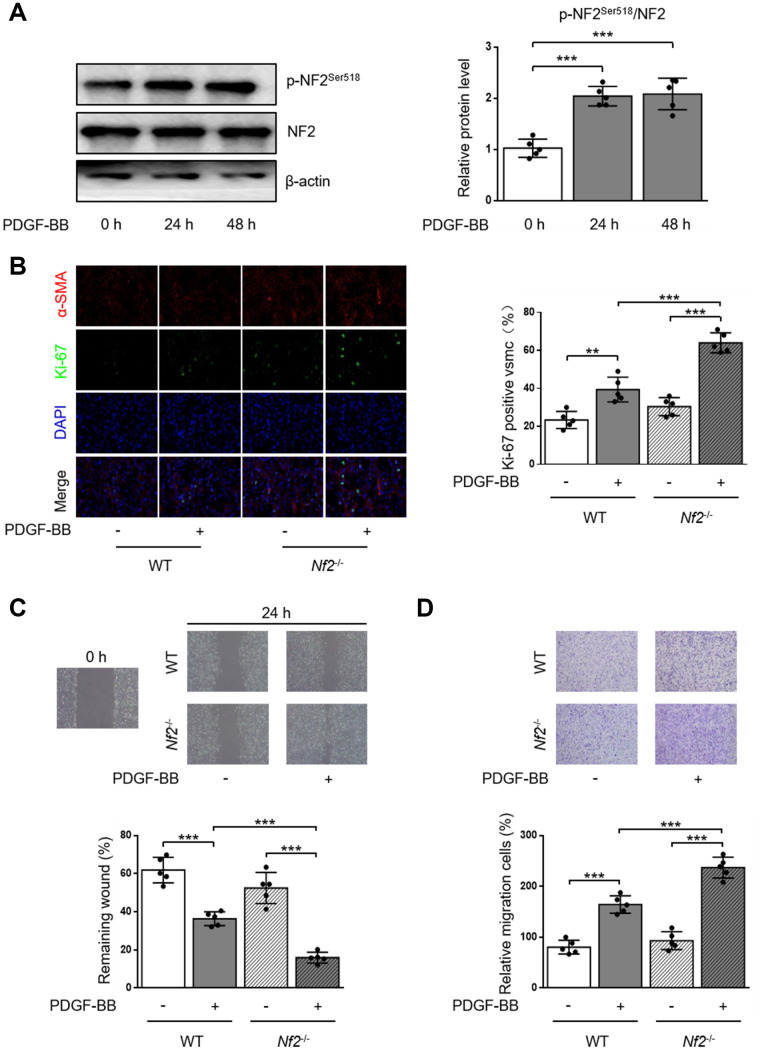
**VSMC proliferation and migration in vitro is elevated after NF2 knockdown.** (**A**) The relative protein expression levels of p-NF2^Ser518^ and NF2 were determined by immunoblotting in VSMC after 0, 24 and 48 h of physiological saline or PDGF-BB (30 ng/mL) treatment (n=5). (**B**) VSMC isolated from WT or *Nf2*^-/-^ mice was stained with SM α-actin (red), Ki-67 (green) and DAPI (blue) after 48 h of physiological saline or PDGF-BB (30 ng/mL) treatment. Representative images (left) and corresponding quantification of Ki-67 positive VSMC (right) were shown (n=5). Magnification 400×. (**C**) Migration of VSMC isolated from WT and *Nf2*^-/-^ mice after 24 h of physiological saline or PDGF-BB (30 ng/mL) treatment was measured via wound healing assay. Representative images (upper panel) and corresponding quantification of healing rates (lower panel) were shown (n=5). Magnification 100×. (**D**) VSMC isolated from WT or *Nf2*^-/-^ mice after 8 h of physiological saline or PDGF-BB (30 ng/mL) treatment was assessed by transwell assay. Representative images (upper panel) and corresponding quantification of migration cells (lower panel) were shown (n=5). Magnification 100×. Data are shown as mean ± S.D. **P<0.01 and ***P<0.001 denote statistical comparison between the two marked groups, respectively.

PDGF-BB is a potent stimulator to induce proliferation and migration of VSMC [17, 18]. We first monitored the alteration of p-NF2^Ser518^ after PDGF-BB treatment by using western blot. As the result showed, phosphorylation of NF2 was significantly elevated in VSMC after both 24 and 48 hours of PDGF-BB treatment (Figure 2A). The Ki-67 immunofluorescence assay showed increased Ki-67-positive VSMC which was isolated from WT mice after PDGF-BB treatment. PDGF-BB induced higher level of proliferation after NF2 knockdown (Figure 2B). We next determined whether NF2 was important for VSMC migration induced by PDGF-BB via traditional scratch wound healing assay. As expected, PDGF-BB accelerated wound closure in VSMC isolated from both WT and *Nf2*^-/-^ mice. Additionally, VSMC challenged with NF2 knockdown showed faster rate of wound closure upon PDGF-BB stimulation (Figure 2C). The result of transwell assay further supported this finding. Compared with VSMC isolated from WT mice, VSMC with NF2 knockdown displayed stronger capacity of migration after PDGF-BB treatment (Figure 2D). Therefore, these results imply that NF2 knockdown promotes excessive VSMC proliferation and migration induced by PDGF-BB.

### NF2 phosphorylation and subsequent nuclear import of YAP in VSMC following PDGF-BB or injury stimulation

**Figure 3:**
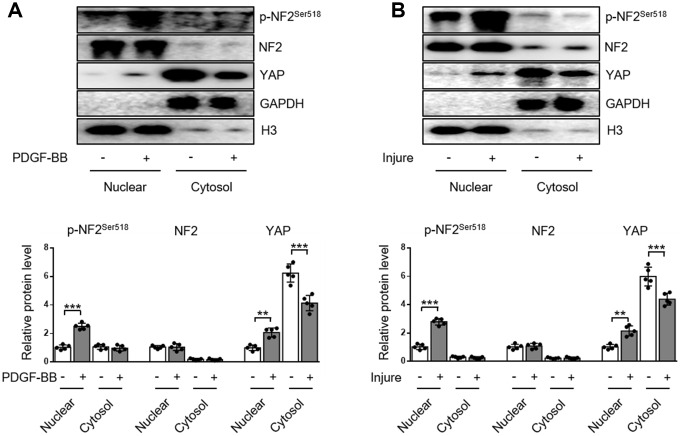
**Enhanced NF2 phosphorylation and subsequent nuclear translocation of YAP following PDGF-BB treatment or injury.** (**A**) Nuclear and cytosolic-enriched fractions were prepared from VSMC, which was treated by PDGF-BB (30 ng/mL) for 48 h. The relative protein expression levels of p-NF2^Ser518^, NF2 and YAP were determined by immunoblotting (n=5). (**B**) Nuclear and cytosolic-enriched fractions were prepared from arteries of C57BL/6J mice at day 28 after vascular injury. The relative protein expression levels of p-NF2^Ser518^, NF2 and YAP were determined by immunoblotting (n=5). Data are shown as mean ± S.D. **P<0.01 and ***P<0.001 denote statistical comparison between the two marked groups, respectively.

The subcellular distributions of NF2 include nucleus, cytosol, tight junctions, adherens junctions, etc [9]. In the current study, we demonstrated the distribution of NF2 in VSMC or artery was more abundant in nucleus than in cytosol. Neither PDGF-BB nor injury affected the subcellular translocation of NF2 (Figure 3A and 3B). Interestingly, Detection of p-NF2 revealed that PDGF-BB-induced enhanced NF2 phosphorylation in VSMC was more pronounced at the nucleus, while cytosolic p-NF2 did not show obvious alteration after PDGF-BB treatment (Figure 3A). We also tested the nuclear and cytosolic localization of p-NF2 in arteries. In consistent with in vitro result, NF2 phosphorylation in artery was significantly elevated by injury in nucleus instead of cytoplasm (Figure 3B).

YAP is an important inducer of VSMC proliferation and migration [12, 13]. Since NF2 promotes translocation of nuclear YAP to cytosol in cardiomyocytes [9], we also investigated the subcellular location of YAP in VSMC. After PDGF-BB stimulation, nuclear YAP was significantly elevated, while cytosolic YAP declined, indicating a nuclear import of YAP caused by PDGF-BB (Figure 3A). Simultaneously, injury in vivo also blocked cytosolic translocation of YAP (Figure 3B). The alteration of subcellular YAP in VSMC was also consistent with enhanced NF2 phosphorylation according to a recent study [9]. These results reveal that increased phosphorylation of nuclear NF2 may induce re-distribution of cellular YAP to promote VSMC proliferation, migration and neointima hyperplasia in response to injury.

### NF2 ablation promotes VSMC proliferation, migration and neointima hyperplasia via reducing phosphorylation of YAP

**Figure 4:**
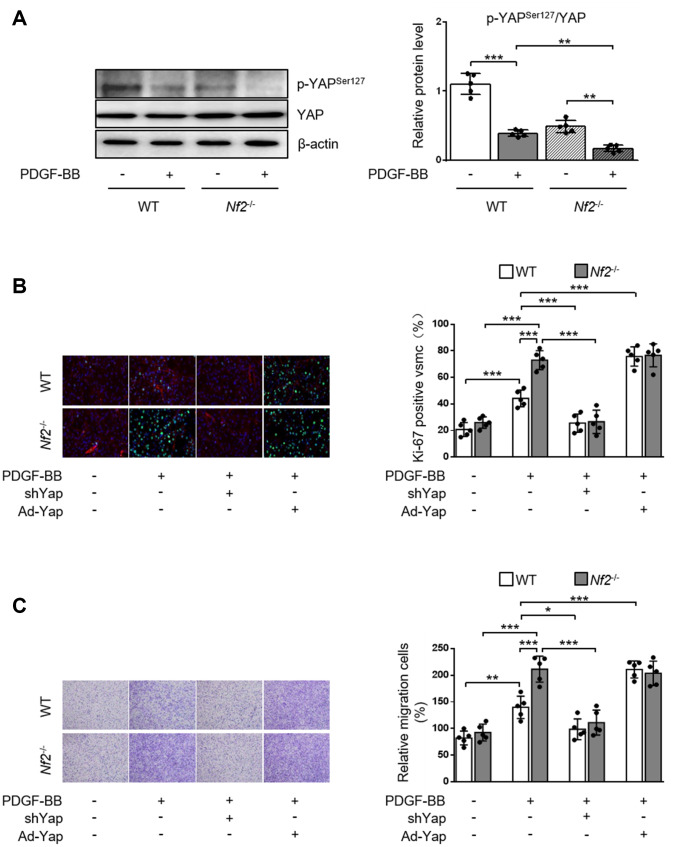
**NF2 knockdown accelerates PDGF-BB-induced VSMC proliferation and migration in a YAP-dependent manner. VSMC isolated from WT or *Nf2*^-/-^ mice was transfected by shCon, shYap, Ad-Con and Ad-Yap.** Then the VSMC received 48 h of physiological saline or PDGF-BB (30 ng/mL) treatment. (**A**) The relative protein expression levels of p-YAP^Ser127^ and YAP were determined by immunoblotting in VSMC treated as above mentioned (n=5). (**B**) VSMC treated as above mentioned was stained with SM α-actin (red), Ki-67 (green) and DAPI (blue). Representative images (left) and corresponding quantification of Ki-67-positive VSMC (right) were shown (n=5). Magnification 400×. (**C**) VSMC treated as above mentioned was assessed by transwell assay. Representative images (left) and corresponding quantification of migration cells (right) were shown (n=5). Magnification 100×. Data are shown as mean ± S.D. *P<0.05, **P<0.01 and ***P<0.001 denote statistical comparison between the two marked groups, respectively.

NF2 can modulate the activity of YAP in various organs such as heart [9], liver [19] and brain [20]. We investigated whether NF2 regulated YAP activity in VSMC and vessels. Previous study showed that phosphorylation of YAP blocked its nuclear translocation and thus inhibited YAP-dependent transcriptional regulation of genes [11]. Therefore, we determined whether NF2 knockdown altered the expression of p-YAP in VSMC. Loss of NF2 in VSMC caused significant decrease in phosphorylation of YAP at site Ser127. Simultaneously, PDGF-BB led to a further decrease in YAP phosphorylation upon NF2 knockdown (Figure 4A). Furthermore, Declined YAP phosphorylation induced by NF2 knockdown existed in both nucleus and cytoplasm after PDGF-BB treatment (Supplementary Figure 5). After NF2 knockdown, nuclear YAP was significantly upregulated, while cytosolic YAP was downregulated. The alteration of YAP phosphorylation induced by NF2 knockdown was also consistent with subcellular re-distribution of YAP. Since dephosphorylation of YAP is known to induce nuclear import of YAP, we utilized Yap shRNA and Ad-Yap to modulate YAP content and explored whether altered expression of p-YAP could explain the promotive effect of NF2 knockdown on VSMC proliferation and migration. Ablation of YAP abolished excessive VSMC proliferation induced by both NF2 knockdown and PDGF-BB stimulation. Overexpression of YAP significantly increased PDGF-BB-induced VSMC proliferation. Additionally, NF2 knockdown failed to further enhance PDGF-BB-induced VSMC proliferation on the basis of YAP overexpression (Figure 4B). Transwell assay showed that YAP inhibition abolished PDGF-BB-mediated excessive VSMC migration, while YAP overexpression caused a further increase in the rate of VSMC migration. More importantly, the capacity of NF2 knockdown to further promote VSMC migration disappeared after both ablation and overexpression of YAP (Figure 4C). Therefore, NF2 knockdown accelerates VSMC proliferation and migration via reducing expression of p-YAP.

**Figure 5:**
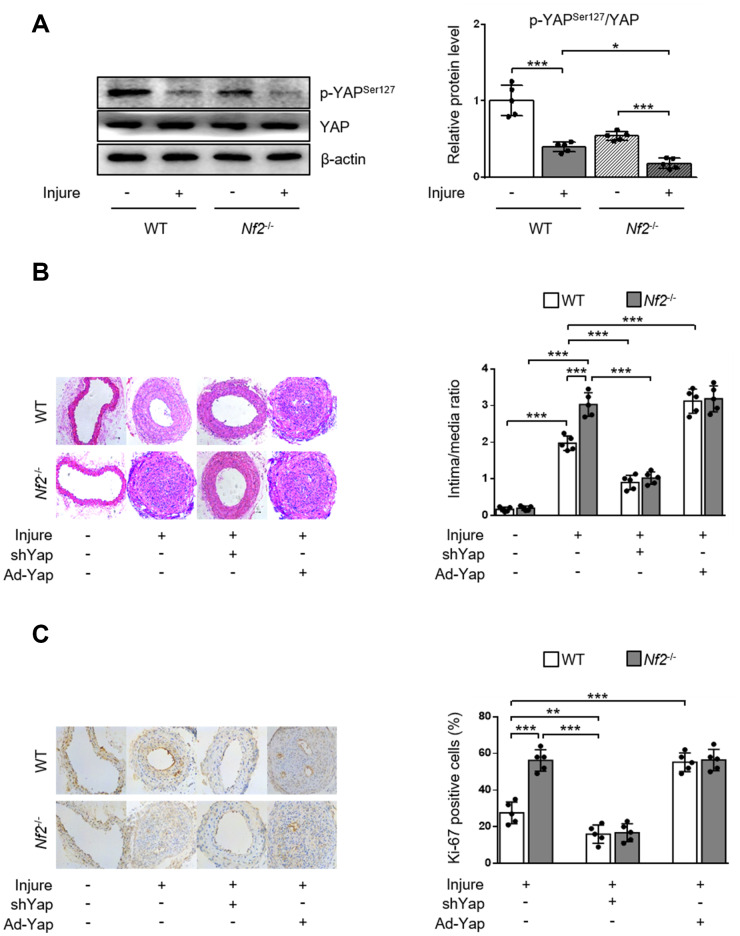
**YAP is required for NF2 knockdown-mediated neointima hyperplasia after vascular injury.** WT and *Nf2*^-/-^ mice received injection of shCon, shYap, Ad-Con and Ad-Yap into the injured left common carotid artery via the external carotid artery immediately after injury and then incubated for 30 min. The mice subsequently received intravenous injection of these adenovirus via tail vein at 7, 14, 21 days after injury. (**A**) The relative protein expression levels of p-YAP^Ser127^ and YAP were determined by immunoblotting in arteries of WT and *Nf2*^-/-^ mice at day 28 after vascular injury (n=5). (**B**) Representative H&E staining of carotid arteries from WT or *Nf2*^-/-^ mice treated as above mentioned (left) and corresponding quantification for ratio of intima/media (right) were shown (n=5). Magnification 200×. (**C**) Immunohistochemistry staining of Ki-67 (brown) in sections of carotid arteries from WT or *Nf2*^-/-^ mice treated as above mentioned (left) and corresponding quantification for Ki-67-positive cells within neointima (right) were shown (n=5). Magnification 200×. Data are shown as mean ± S.D. *P<0.05, **P<0.01 and ***P<0.001 denote statistical comparison between the two marked groups, respectively.

To validate the role of YAP activity in NF2 knockdown-mediated promotion of neointima hyperplasia after vascular injury in vivo, we first determined phosphorylation of YAP via performing wire injury in carotid arteries of WT and *Nf2*^-/-^ mice. After injury, phosphorylation of YAP significant decreased in injured arteries of WT mice. The injured arteries in *Nf2*^-/-^ mice exhibited further decrease in the expression of p-YAP (Figure 5A). Since dephosphorylation of YAP by NF2 knockdown led to increased nuclear YAP content, we used adenovirus carrying shYap or Ad-Yap to achieve regulation of YAP expression. Consistent with our findings in vitro, *Nf2*^-/-^ mice had significantly increased intima/media ratio and Ki-67-positive area after injury compared to WT mice. Interestingly, there were no differences in intima/media ratio and Ki-67-positive area between WT and *Nf2*^-/-^ mice after injury upon ablation or overexpression of YAP (Figure 5B and 5C). Collectively, these results indicate that declined YAP phosphorylation is responsible for NF2 knockdown-mediated promotion of VSMC proliferation, migration and neointima hyperplasia after vascular injury.

### Enhanced interaction between YAP and TEAD1 is required for NF2 knockdown-mediated promotive effect on VSMC proliferation and migration

**Figure 6:**
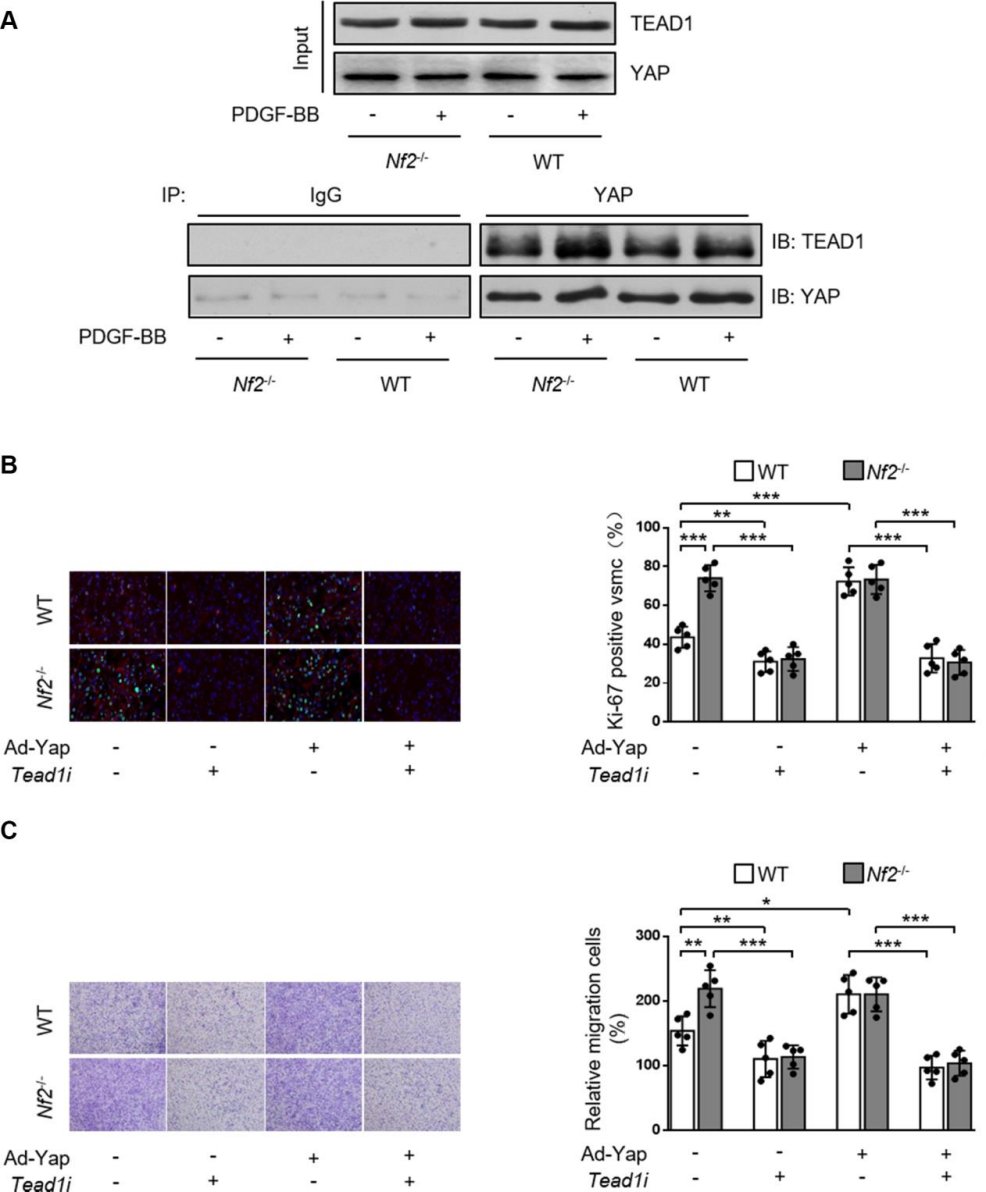
**NF2 knockdown causes increased VSMC proliferation and migration induced by PDGF-BB via inducing YAP-TEAD1 interaction.** (**A**) VSMC isolated from WT or *Nf2*^-/-^ mice after 48 h of physiological saline or PDGF-BB (30 ng/mL) treatment was subjected to immunoprecipitation using anti-YAP antibody or control IgG. Inputs and immunocomplexes were analyzed by immunoblotting. VSMC isolated from WT and *Nf2*^-/-^ mice was transfected with control siRNA, *Teadi* or Ad-Con, Ad-Yap and then treated by physiological saline or PDGF-BB (30 ng/mL) for 48 h. (**B**) VSMC treated as above mentioned was stained with SM α-actin (red), Ki-67 (green) and DAPI (blue). Representative images (left) and corresponding quantification of Ki-67-positive VSMC (right) were shown (n=5). Magnification 400×. (**C**) VSMC treated as above mentioned was assessed by transwell assay. Representative images (left) and corresponding quantification of migration cells (right) were shown (n=5). Magnification 100×. Data are shown as mean ± S.D. *P<0.05, **P<0.01 and ***P<0.001 denote statistical comparison between the two marked groups, respectively.

**Figure 7:**
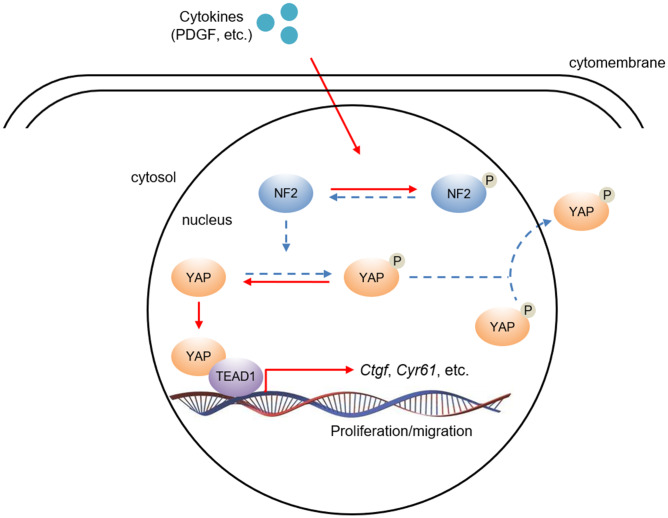
**The hypothesis of NF2/YAP/TEAD1 signaling axis in VSMC after PDGF-BB stimuli.** PDGF-BB excretion after vascular injury induces nuclear NF2 phosphorylation, thereby causing inactivation of NF2. Decreased active NF2 led to dephosphorylation of YAP and subsequent nuclear translocation of YAP. Increased nuclear YAP binds to TEAD1 and positively regulates relative target gene expression.

Current literature suggest a crucial role of transcription co-factor YAP in regulating downstream signaling transduction via binding with other transcription factors [21, 22]. TEAD promotes VSMC proliferation, migration [21, 23, 24] and is involved in several vascular diseases such as hypertensive vascular remodeling [25]. Therefore, we investigated whether NF2 functioned via mediating interaction between YAP and TEAD1 in VSMC. Both PDGF-BB stimulation (Figure 6A) and injury (Supplementary Figure 6) induced increased interaction between YAP and TEAD1. In parallel, we found that *Nf2*^-/-^ mice exhibited further increase in YAP-TEAD1 interaction compared to WT mice after PDGF-BB treatment (Figure 6A) or injury (Supplementary Figure 6). Next, we utilized *Tead1i* to validate the role of YAP-TEAD1 interaction in NF2 knockdown-mediated promotion of VSMC proliferation and migration. As the result showed, inhibition of TEAD1 blocked PDGF-BB-induced excessive VSMC proliferation and migration (Figure 6B and 6C). Simultaneously, both NF2 knockdown and YAP overexpression failed to abolish the suppressive effects of TEAD1 inhibition on proliferation and migration of VSMC (Figure 6B and 6C). Taken together, these results strongly suggest that enhanced YAP-TEAD1 interaction is responsible for NF2 knockdown-mediated promotion of VSMC proliferation and migration (Figure 7).

## DISCUSSION

Originally recognized as a tumor suppressor, NF2 is recently confirmed to be associated with cardiovascular disorders [9]. A recent study has demonstrated that NF2 is critical in regulation of cardiac ischemic/reperfusion injury via modulating hippo pathway [9]. Mutation or deletion of *NF2* in patients can lead to arterial dysplasia or aneurysm [26–28]. NF2 can also regulate formation of neovascularization in tumor via semaphorin 3F [29]. The above-mentioned studies suggest that NF2 plays a critical role in regulation of vascular structure and function. However, the roles and mechanisms of NF2 in neointima hyperplasia and vascular restenosis after mechanical injury have not been explored. In the current study, we identified NF2 as a novel inhibitor in VSMC proliferation, migration and vascular restenosis. p-NF2 was elevated during VSMC proliferation/ migration induced by PDGF-BB in vitro or vascular injury in vivo. NF2 knockdown caused aggravated neointima hyperplasia in response to injury through suppression of YAP phosphorylation and subsequent promotion of YAP-TEAD1 interaction.

There is a dynamic balance between proliferation and apoptosis in VSMC under physiological conditions [30]. Certain pathological conditions, including mechanical injury and abnormal secretion of growth factors, will break this equilibrium and induce excessive proliferation and migration of VSMC [30, 31]. Various signaling pathways such as Mitogen Activated Protein Kinases, Phosphatidylinositol-3-Kinase/Akt/Mammalian Target of Rapamycin Complex 1 (mTORC1) are involved in VSMC proliferation and migration [32]. However, the morbidity of vascular restenosis is still close to 10% after traditional treatments based on above pathways [33]. Therefore, it is critical to search for novel targets inhibiting VSMC proliferation. PDGF is a major player in VSMC proliferation, migration and neointima hyperplasia after vascular injury [34]. Treatment of PDGF-BB in VSMC is known to activate cyclic adenosine monophosphate/protein kinase A (cAMP/PKA) pathway [35], which negatively regulates NF2 activity via inducing phosphorylation on Ser518 [36]. Additionally, NF2 is also involved in modulation of growth-associated pathways such as mTORC1 [37] and hippo pathway [9], which are critical in regulating VSMC proliferation and migration [7]. These studies remind us that NF2 may be an important suppressor of VSMC proliferation and migration. This hypothesis is also confirmed by the promotive effects of NF2 knockdown on VSMC proliferation and migration in our current study.

The subcellular localization of NF2 is cell type-specific and has important cellular functions [9]. Association of NF2 with plasma membrane and binding of NF2 to Lats1/2 are required for engagement of downstream hippo signaling and subsequent YAP phosphorylation [38]. NF2 can also shift to nucleus and suppress mitosis via inhibiting *Crl4* (DCAF1) [39]. These two studies indicate that NF2 may exert different roles based on its different subcellular location. We observed obviously higher protein levels of NF2 and p-NF2 in the nucleus of VSMC and carotid artery than cytosol. Both nuclear and cytosolic NF2 were not affected by PDGF-BB or injury. Importantly, PDGF-BB or injury caused significantly elevated expression of p-NF2 in nucleus rather than cytosol. Since phosphorylation at site Ser518 induces inactivation of NF2, we propose that declined nuclear active NF2 is the focal issue resulting in abnormal VSMC proliferation, migration and neointima hyperplasia after injury.

Another significance of this study is that we characterized YAP as the downstream mediator of NF2 signaling in VSMC. YAP transfers from nucleus to cytosol via phosphorylation by NF2 and causes declined transcription of downstream genes such as *Ctgf* and *Cyr61*, while NF2 mutation or inactivation induces YAP dephosphorylation and subsequent nuclear import of YAP [9, 11]. We observed increased nuclear YAP and declined cytosolic YAP followed by PDGF-BB treatment or injury. NF2 knockdown further reduced phosphorylation of YAP, indicating the elevated nuclear phosphorylation or inactivation of NF2 was responsible for PDGF-BB- or injury-induced YAP dephosphorylation and translocation of YAP from cytosol to nucleus. YAP has been shown to be crucial for vascular development [40], angiogenesis [41], endothelial function and vascular inflammation [42], as well as promotion of VSMC proliferation and migration [12, 13]. On the contrary, strategies to reduce YAP expression and activity attenuate VSMC proliferation, migration and protect against atherosclerosis and neointima formation [14, 43]. We demonstrated that the promotion of VSMC proliferation, migration and vascular restenosis in *Nf2*^-/-^ mice was abrogated by YAP ablation. Compared to WT mice, *Nf2*^-/-^ mice failed to further aggravate neointima hyperplasia after injury upon YAP overexpression. These data provide strong evidence that YAP acts downstream of NF2 to promote vascular restenosis.

It is known that YAP binds with other transcription factors to modulate expression of downstream genes [21, 22]. Among these transcription factors, YAP exerts its growth promoting property mainly by binding to TEAD [44]. For example, Ablation of YAP inhibits expression of numerous YAP-inducible genes and largely blocks YAP-mediated overgrowth phenotype [44]. The phenotype of the *Tead1/2*-knockout mice is similar to YAP-knockout mice [45]. In the current study, we found that YAP directly bound to TEAD1 in VSMC. More importantly, NF2 knockdown further enhanced the binding between YAP and TEAD1. Previous studies have demonstrated that TEAD1 is crucial in promoting VSMC proliferation. On one hand, TEAD1 alone promotes VSMC proliferation via upregulating glutamine uptake. On the other hand, TEAD1 can also bind to YAP/TAZ to induce mitogenesis in VSMC [21, 24]. In agreement, we found that inhibition of TEAD1 abolished PDGF-BB-mediated excessive VSMC proliferation and migration, which were not rescued by NF2 knockdown or YAP overexpression. Therefore, we conclude that TEAD1 serves as the core downstream signal of NF2/YAP axis in the development of vascular restenosis.

## MATERIALS AND METHODS

### Animals

C57BL/6J mice were obtained from the Dashuo Animal Science and Technology (Chengdu, Sichuan, China). We generated VSMC-specific *Nf2*-knockdown mice by crossing mice carrying floxed *Nf2* alleles (*Nf2*^fl/fl^) with sm22cre mice. By using mixed strain *Nf2*^fl/fl^sm22cre^-/-^ and C57BL/6 *Nf2*^WT/WT^sm22cre^+/+^ purchased from Nanjing Biomedical Research Institute of Nanjing University (Nanjing, Jiangsu, China), we firstly generated F1 *Nf2*^fl/WT^sm22cre^+/-^ mice. Then the F1 progeny was further self-crossed to finally obtain male *Nf2*-loss mice with *Nf2*^fl/fl^sm22cre^+/-^ genotype (*Nf2*^-/-^) for experiments. Littermate male mice (*Nf2*^WT/WT^sm22cre^+/-^) were used as controls (WT). All experiments were approved by the Institutional Animal Care and Use Committee and the Ethic Committee of The General Hospital of Western Theater Command. The mice (8-10 weeks old) were housed as following described: 12-hour light/dark cycle, 22-25°C, free access to food and water and periodic air changes. The injury of left common carotid artery was conducted as our previous study described [46]. Briefly, WT and *Nf2*^-/-^ mice (8-10 weeks old) were firstly anesthetized with pentobarbital (40 mg/kg, intraperitoneal). Then a small midline incision in the neck area was performed and the left common carotid artery was isolated. The proximal common carotid artery and internal carotid artery were temporarily occluded with clamps. Next, the external carotid artery was ligated. A 0.38-mm guidewire was inserted into the common carotid artery and passed 3 times with rotation backward and forward to denude the endothelium. The vascular clamps were then removed to restore the blood flow. The incision of neck was finally closed. Mice were deeply anesthetized with pentobarbital (100 mg/kg) and then followed by decapitation for further experiments.

### Immunoprecipitation and Western blot analysis

For immunoprecipitation assay, artery homogenates or VSMC extracts were prepared in lysis buffer (50 mmol/L Tris·HCl (pH 7.5), 150 mmol/L NaCl, 0.5% IGEPAL CA-630, 0.5% deoxycholic acid, 0.1% SDS, 1 mmol/L EDTA, 1 mmol/L NaF, 0.1 mmol/L Na_3_VO_4_, 50 μmol/L phenylmethylsulfonyl fluoride, 5 μg/mL leupeptin, 5 μg/mL aprotinin). Samples were then incubated with primary antibody at 4°C overnight, and immunocomplexes were precipitated after 1 hour of incubation with sepharose A/G beads (Santa Cruz, Delaware, CA). Western blot analysis was performed as described previously [46]. Extraction of artery and VSMC were lysed with RIPA buffer (Beyotime Institute of Biotechnology, Shanghai, China). Antibodies against phospho (p)-NF2^Ser518^, NF2, p-YAP^Ser127^, YAP, TEAD1, GAPDH, H3, β-actin were purchased from Cell Signaling Technology (Danvers, MA). The luminescent signal was determined by exposure to x-ray film and quantitative analysis was completed with Image J software (Bethesda, MA).

### Real time quantitative PCR (qRT-PCR)

Total RNA was extracted by using Trizol agent (Life Technologies, Carlsbad, CA) as previously described [46]. Briefly, RNA (1 μg) was reverse-transcribed into cDNA through a Bestar qPCR RT Kit (DBI Bioscience, Ludwigshafen, RP). The reaction of real time qRT-PCR was performed in ABI Prism 7700 Sequence Detector (Applied Biosystems, Carlsbad, CA). *Actb* was utilized as a housekeeping gene and the classical ΔΔCt method was used for normalization of gene expression. Primers used for all genes were listed as following: *Nf2* (F, CGCCAAGTCCCGAGTGG; R, AACAAGCCAGCCCTCTACTG), *Yap* (F, GAAACCTCCTCCCGTGTCTG; R, GCTCAGTCCAACTACCCCAC), *Actb* (F, TCCTTCTTGGGTATGGAA; R, AGGAGGAGCAATGATCTTGATCTT), *α-SMA* (F, GTCCCAGACATCAGGGAGT-AA; R, TCGGATACTTCAGCGTCAGGA) [16], *Calponin* (F, TCTGCACATTTTAACCGAGGTC; R, GCCAGCTTGTTCTTTACTTCAGC) [16], *SM-MHC* (F, AAGCTGCGGCTAGAGGTCA; R, CCCTCCCTTTGATGGCTGAG) [16].

### Immunohistochemistry

Histological analyses of carotid arteries were conducted by standard hematoxylin and eosin (H&E) and Ki-67 staining as previously described [46]. For H&E staining, the artery sections (4-5 μm) were stained with hematoxylin and eosin. For Ki-67, p-NF2^Ser518^ and NF2 staining, the artery sections were firstly incubated with primary antibody against Ki-67, p-NF2^Ser518^ and NF2 (Cell Signaling Technology, Danvers, MA) at 4°C overnight after being blocked, followed by incubation with secondary antibody and finally counterstained with mayer hematoxylin. The investigators were blinded for acquiring the images. Acquired images were determined by utilizing Image-Pro Plus software (Bethesda, MD).

### Isolation, culture and treatment of VSMC

VSMC was obtained from thoracic aortas of 8- to 10-week-old WT and *Nf2*^-/-^ mice by enzyme digestion method as previously described [46]. Briefly, VSMC was digested with 0.25% trypsin (Beyotime Institute of Biotechnology) and cultured in complete medium [Dulbecco’s modified Eagle’s medium (DMEM; HyClone, Carlsbad, CA) supplemented with 10% fetal calf serum (Invitrogen, Carlsbad, CA), streptomycin (100 mg/mL) and penicillin (100 units/mL)] at 37°C in humidified 5% CO_2_ atmosphere. The VSMC was then centrifugated and cultured in complete medium at 37°C in 5% CO_2_ atmosphere. Recombined human PDGF-BB with a dose of 30 ng/mL (R&D Systems, Minneapolis, MI) was utilized to treat VSMC.

### Immunofluorescent (IF) staining

IF staining was conducted as described previously [46]. Cell plates were firstly washed by PBS for three times, followed by fixation with 4% paraformaldehyde for 20 min and then blocked in 1% blocking solution. The VSMC was then incubated overnight with primary antibodies against SM α-actin and Ki-67 (1: 1000; Cell Signaling Technology) in dark at 4°C. After washed with PBS, VSMC was then incubated with Alexa Fluor 594F(ab’)-conjugated goat anti-mouse and Alexa Fluor 488-conjugated goat anti-rabbit secondary antibodies (1: 2500; Molecular Probes Inc., Eugene, OR), respectively, for 1 h in dark. Subsequently, VSMC was incubated with DAPI (5mg/ml; VECTOR Labs, Burlingame, CA) for 5 s in the room temperature. Images were acquired by using an immunofluorescent microscopy (Leica MPS 60; Wetzlar, HD). The fluorescence intensity was analyzed by using Image J software (Bethesda, MA).

### Wound-healing assay

VSMC was seeded in six-well plates (1 × 10^5^ cells per well) and serum-deprived for 24 h. Subsequently, the VSMC isolated from WT or *Nf2*^-/-^ mice was incubated with physiological saline and PDGF-BB for 24 h. The rates of wound closure were directly evaluated by using microscopic visualization followed with a reference point at the bottom of the wound field, thus permitting photographing of the same spot each time. The remaining cell-free areas were measured at 24 h after injury [46].

### Transwell assay

The migration assay was performed as described previously [46]. VSMC isolated from WT or *Nf2*^-/-^ mice was placed in a upper transwell chamber (Millipore, Darmstadt, HD) with 8-mm pore in each membrane and incubated for 8 h in a 24-well plate (1 × 10^5^ cells per well). The lower chamber was filled with serum-free DMEM with or without PDGF-BB (30 ng/ml). Non-migrated cells were subsequently wiped off from the inside of chamber membrane. VSMC on the lower surface were fixed with 4% paraformaldehyde, washed for 3 times and then stained with 1% crystal violet for 20 min before placed on glass slides. Finally, VSMC in four randomly selected fields per well was counted under a microscope.

### Subcellular fractionation

Extractions of nuclear and cytoplasmic fraction with isolated mouse artery or VSMC were performed as previous research described [9]. Briefly, sample homogenates were placed in Buffer A [10 mmol/L HEPES (pH 7.6), 1.5 mmol/L MgCl_2_, 10 mmol/L NaCl, 0.1% NP40-alternative, 10% glycerol, 0.1 mmol/L Na_3_VO_4_, 5 μg/mL leupeptin, 5 μg/mL aprotinin] for 15 min on ice. Homogenates were then centrifuged at 13000 × *g* at 4°C for 5 min. The supernatant was collected as cytoplasmic fractions. The pellets were resuspended in Buffer B [20 mmol/L Tris (pH 7.6), 3 mmol/L EGTA, 3 mmol/L EDTA, 250 mmol/L NaCl, 1% NP40 alternative, 20 mmol/L β-glycerophosphate, 0.1 mmol/L Na_3_VO_4_, 5 μg/mL leupeptin, 5 μg/mL aprotinin] and incubated for 10 min on ice. The homogenates were centrifuged at 2700 × *g* at 4°C for 5 min. The pellets were resuspended in RIPA buffer, incubated for 10 min on ice and centrifuged at 13000 × *g* for at 4°C 5 min. The supernatant was collected as the nuclear fraction.

### Construction of adenovirus

The recombinant adenovirus encoding Yap shRNA (shYap) or control shRNA (shCon) were constructed according to the manufacturer’s instructions (Genechem, Shanghai, China). The adenovirus expressing Yap (Ad-Yap) and control viruses (Ad-Con) were conducted according to the manufacturer’s instructions (Genechem). For transfer in vitro, VSMC was transfected with adenovirus (3 pfu/cell) for 72 hours. For transfer in vivo, adenovirus (10^11^ pfu/mL) was injected into the injured left common carotid artery via the external carotid artery immediately after injury and then incubated for 30 min. The mice subsequently received intravenous injection of adenovirus via tail vein at 7, 14, 21 days after injury.

### Transfection with siRNA

We performed siRNA transfection using X-tremeGENE siRNA transfection kit (Roche; Basel, BS, Switzerland) according to the manufacturer’s protocol. Briefly, VSMC was transfected with target-specific *Tead1* siRNA (*Tead1i*; GenePharma, Shanghai, China) and incubated with 10 μL X-tremeGENE siRNA transfection reagent. Scramble siRNA was used as a nonspecific control. After 8 hours of transfection, VSMC was refreshed with the complete medium. The sequence of *Tead1i* was shown as following: *Tead1i* (5’-GGCAGATAAGCCGATTGACAACG-3’).

### Statistical analysis

Data are presented as mean ± S.D. Unpaired Student’ *t*-test was used to compare two independent groups. One-way or two-way analysis of variance (ANOVA) was conducted to compare means that involve one or two factors, respectively, with appropriate *post hoc* tests. All tests were two-tailed. *P*<0.05 was considered to be statistically significant.

## Supplementary Material

Supplementary Figures
