# Supplementary material for: NF2 deficiency accelerates neointima hyperplasia following vascular injury via promoting YAP-TEAD1 interaction in vascular smooth muscle cells
Source: Aging (Albany NY). 2020 May 18;12(10):9726–44. doi: 10.18632/aging.103240 (PMC7288949; doi:10.18632/aging.103240)
Supplement: Supplementary Figures [file aging-12-103240-s001..pdf]

SUPPLEMENTARY FIGURES

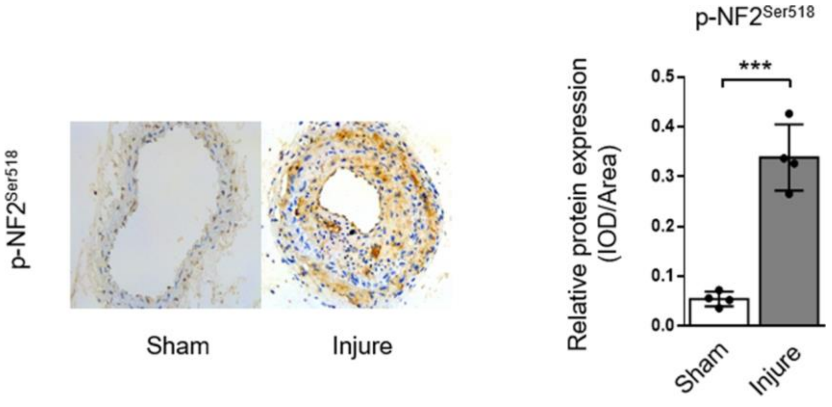

Supplementary Figure 1. Vascular injury induces enhanced phosphorylation of NF2 in neointima. The relative protein expression of p-NF2^{Ser518} by immunohistochemistry in carotid artery of mice at day 28 after sham operation or wire injury (left) and corresponding quantification (right) were shown (n=4). Magnification 200×. Data are shown as mean ± S.D. ***P<0.001 denote statistical comparison between the two marked groups, respectively.

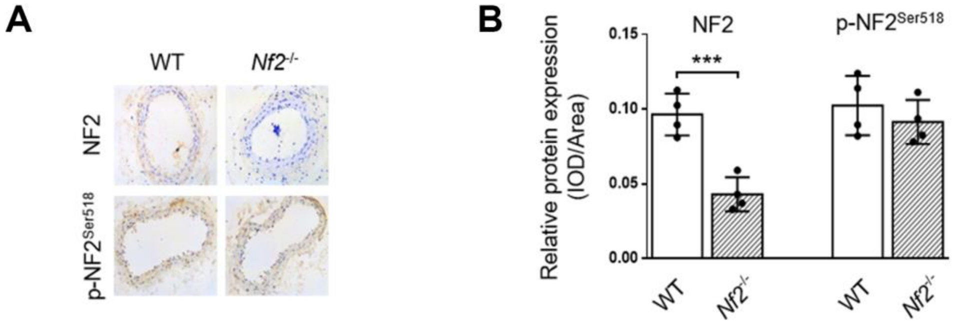

Supplementary Figure 2. The relative protein expression levels of NF2 and p-NF2^{Ser518} by immunohistochemistry in WT or *Nf2*$^{-/-}$ mice at the age of 10 weeks. The representative picture (**A**) and corresponding quantification (**B**) for NF2 and p-NF2^{Ser518} were shown (n=4). Magnification 200×. Data are shown as mean ± S.D. **P<0.01 and ***P<0.001 denote statistical comparison between the two marked groups, respectively.

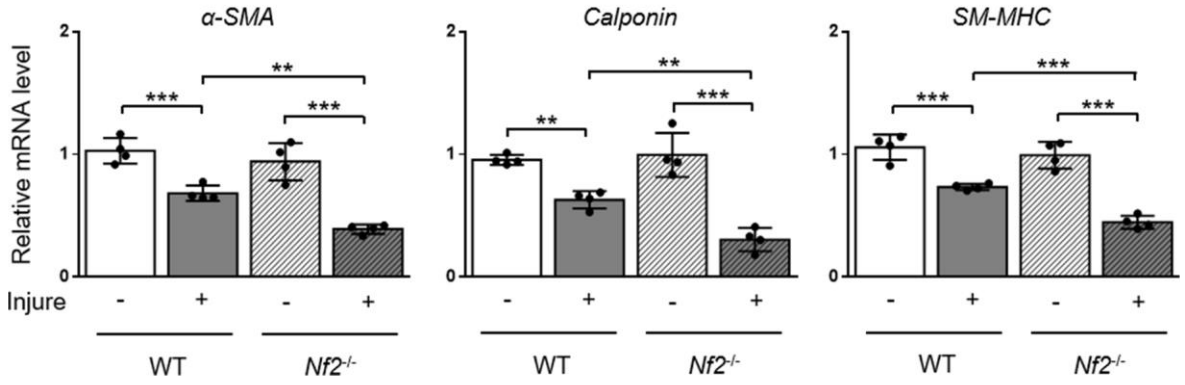

Supplementary Figure 3. The mRNA levels of differentiation marker genes decline after injury in mice deficient for *Nf2*. The mRNA levels of *α-SMA*, *Calponin* and *SM-MHC* in carotid arteries from WT or *Nf2*$^{-/-}$ mice at day 28 after sham operation or injury (n=4). Data are shown as mean ± S.D. ***P*<0.01 and ****P*<0.001 denote statistical comparison between the two marked groups, respectively.

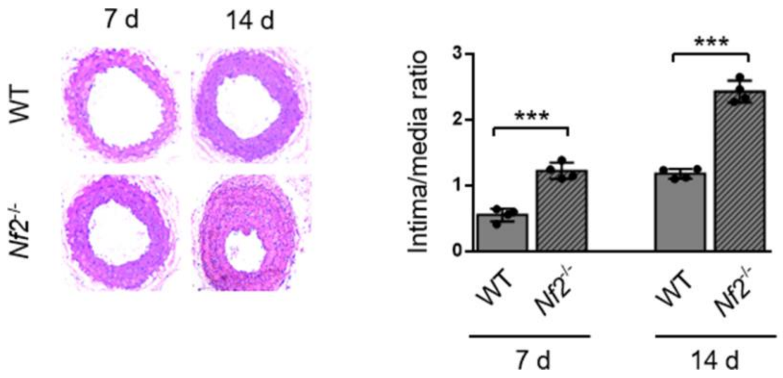

Suppleemntary Figure 4. NF2 knockdown also enhances injury-induced neointima hyperplasia at early and midway stage. Representative H&E staining of carotid arteries from WT or *Nf2*$^{-/-}$ mice at day 7 or 14 after wire injury (left) and corresponding quantification for ratio of intima/media (right) were shown (n=4). Magnification 200×. Data are shown as mean ± S.D. ****P*<0.001 denote statistical comparison between the two marked groups, respectively.

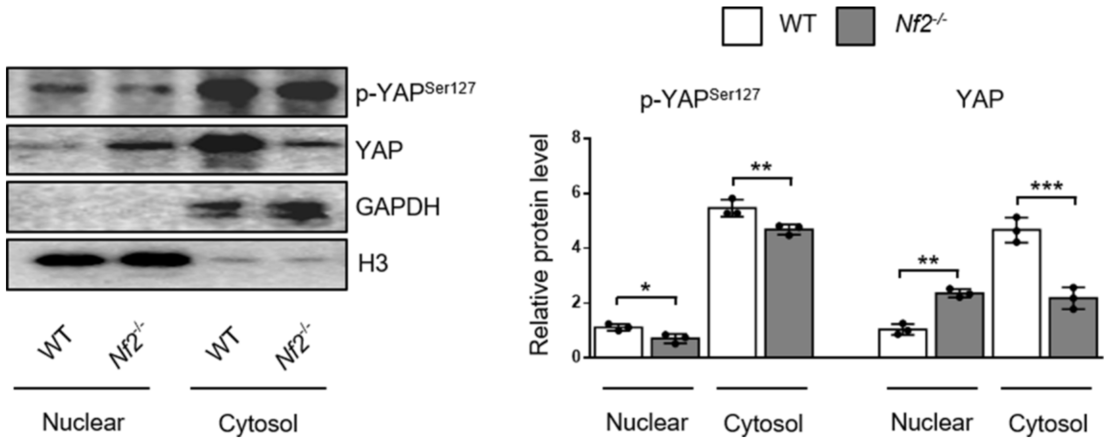

Supplementary Figure 5. NF2 knockdown causes declined YAP phosphorylation in both nucleus and cytoplasm of PDGF-BB-treated VSMC. VSMC from WT or *Nf2*$^{-/-}$ mice was treated by PDGF-BB (30 ng/mL) for 48 h. The nuclear and cytosolic-enriched fractions were then prepared. The relative protein expression levels of p-YAP^{Ser127} and YAP were determined by immunoblotting (n=3). Data are shown as mean ± S.D. *P<0.05, **P<0.01 and ***P<0.001 denote statistical comparison between the two marked groups, respectively.

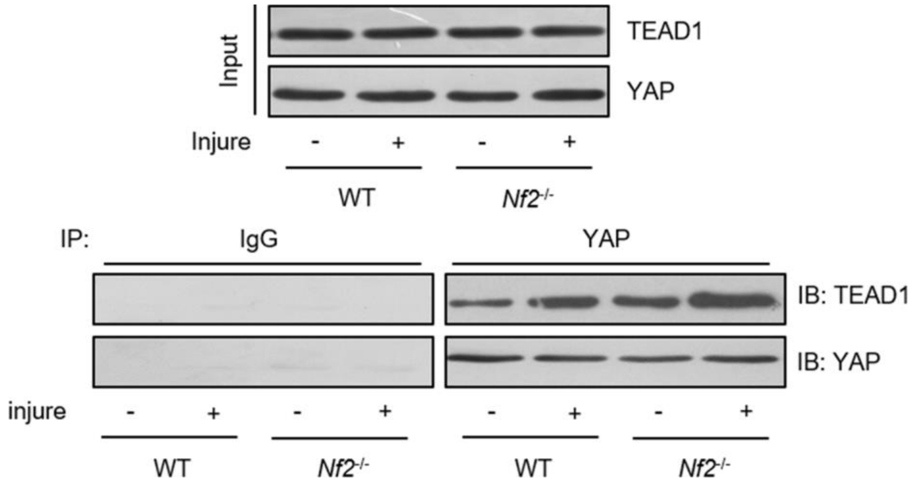

Supplementary Figure 6. NF2 knockdown enhances YAP-TEAD1 interaction in injured carotid artery. Common carotid arteries from WT and *Nf2*$^{-/-}$ mice at day 28 after injury were subjected to immunoprecipitation using anti-YAP antibody or control IgG. Inputs and immunocomplexes were analyzed by immunoblotting.
